# Patient-centered care: achieving higher quality by designing care through the patient’s eyes

**DOI:** 10.1186/s13584-021-00459-9

**Published:** 2021-03-05

**Authors:** Susan Edgman-Levitan, Stephen C. Schoenbaum

**Affiliations:** 1 John D. Stoeckle Center for Primary Care Innovation, Massachusetts General Hospital, Boston, MA USA; 2 Josiah Macy Jr. Foundation, New York, NY USA

## Abstract

Patient centered care requires that health care organizations and health care professionals actively understand what patients value. Fortunately, there are methods for gaining that understanding. But, they need to be adopted much more widely, and patients need to be treated as full partners in their care.

In 2020, the Israel Journal of Health Policy Research published seven papers related to aspects of patient centered care. Several of them highlighted specific strategies that were associated with, or that would improve, patient centered care. This commentary strives to put those papers and their strategies into perspective. Fortunately, the same strategies that lead to excellent patient-centered care lead to health care organizations and settings that are high performing overall, have excellent health outcomes, are safe and desirable workplaces, and are financially strong.

In 1988 the Picker Commonwealth Program for Patient-Centered Care’s research program, based at Boston’s Beth Israel Deaconess Hospital, conducted national qualitative research to identify the aspects of care most valued by patients to guide the development of patient experience surveys that could be used to evaluate care. Patient-centeredness was defined as “Health care that establishes a *partnership among practitioners, patients, and their families*…to ensure that decisions *respect patients’ wants, needs, and preferences* and that patients have the *education and support* they need to *make decisions and participate* in their own care.” [1] Surprisingly, patients never once mentioned the amenities such as parking and food as important, even though most surveys at the time only focused on those topics.

The Picker/Commonwealth work and later the Picker Institute’s research confirmed that patients are often the only ones who can evaluate many aspects of the care and its quality. It doesn’t matter to the patient what providers think they have communicated or documented in the chart. If the patient doesn’t understand or remember it, the quality of care suffers. The Picker approach to measuring the patient’s experience of care, as opposed to their satisfaction with care, has continued through the work of the US Consumer Assessment of Healthcare Providers and Systems (CAHPS) Consortium, funded by the Agency for Healthcare Research and Quality. That work has developed and implemented national standardized patient experience surveys in the US that have been in use since the mid-1990s. These surveys are used by NCQA (the National Committee for Quality Assurance) as part of health plan accreditation [2] and by the Centers for Medicare and Medicaid Services in their value-based payment programs to financially reward higher performing hospitals [3]. These surveys and related measures of quality (e.g., HEDIS) have resulted in steady improvements in the scores [4, 5] and the evolution of a strong array of improvement interventions [6].

In 2001, the United States Institute of Medicine, now the National Academy of Medicine, issued its landmark report, “Crossing the Quality Chasm.” [7] It called for a transformation of the US healthcare system to be guided by 6 aims: Safety, Effectiveness, Efficiency, Patient-Centeredness, Timeliness, and Equity. The report adopted the Picker definition of patient-centered care. Since its publication, many healthcare organizations, both public and private have worked to improve these aims with varying success. Overall, the quality of care in the U.S., and other countries, falls short of what most persons believe it to be and expect that they will experience.

Many organizations have failed to understand that patient-centeredness is the overarching aim that encompasses the rest. The *raison d’etre* for health care and health care systems is to improve the health and well-being of individuals and populations. This requires the patient to be a partner and co-designer of all improvement activities, a concept that many leaders and clinicians still find threatening or unnecessary. In addition to co-designing care, attaining a patient-centered health care system requires an organizational focus on leadership values, human resources policies that recruit and retain staff with aptitudes for service and empathy, and continuous measurement of the patient experience using both well designed surveys that measure the aspects of care that patients care about and qualitative methods to help collect improvement ideas. These same foundational strategies create high performing organizations that are safe, excellent workplaces, and financially strong.

Health care organizations around the world tend to focus on the needs of their physicians and staff, rather than on their patients. Almost all of them believe that they are contributing to the health and well-being of the public and individual patients. But unless they are actively partnering with their patients to understand what matters most, they will not achieve the outcomes of care they desire. Clinicians often assume that they understand the experience of illness [8]; but knowledge about physiology or diagnosis and treatment are not the same as understanding how it feels to be sick or to manage a chronic health problem. Clinicians rarely experience the frustrations and challenges of getting care until they or a family member needs care. Patients bring the lived experience, as well as knowledge about how well the healthcare system functions to meet their needs. It is not uncommon for clinicians and administrators to think that they can speak for their patients because they all are patients or have family members who are. This is a cardinal error. In order to provide truly excellent health care, we must have the input of patients, many patients, because no one person can speak for the experiences of all patients.

Collecting patient experience data over time has reinforced the understanding of how important it is to partner with patients and their families to design care. For decades, the US Institute for Patient and Family Centered Care has promoted patient/family partnerships and developed excellent resources to guide these efforts. Adoption of these concepts is now much more widespread. For example, the state of Massachusetts legislates that all hospitals establish Patient Family Advisory Councils. But myths still abound regarding the value of and barriers to partnerships of this type. These include fear of organizational leaders about “showing our dirty laundry,” concern about inappropriate expectations, worry that clinicians and staff will be subjected to anger or criticisms, and assumptions that patients will have nothing to add. Patients and families fear their recommendations will be ignored or that they will be intimidated by clinicians speaking in jargon. When patients and the clinicians with whom they partner are trained to work collaboratively, these experiences rarely occur.

The widespread adoption of Patient Family Advisory Councils in Massachusetts has helped to identify strategies and approaches that fix problems and are frequently much cheaper and easier to implement than what professionals would design on their own. When a hospital team discharges a patient, having the patient “teach back” the discharge plan is the surest way the team knows that the patient understands what to do, how to do it, and when to do it [9]. Similarly, in ambulatory care, there are significant gaps in patients’ understanding of why they are taking each prescribed medicine, when and how they should take it. “Teach back” is not commonly used and should be. Indeed, not surprisingly, a major reason for patients not taking prescribed medications is not having understood and not having agreed to the need for taking them. GI doctors now realize that there is a reason patients do not remember their post-colonoscopy instructions or when to expect their results. It is not a good idea to give people important information or instructions after they have received drugs such as midazolam.

In 2020, the Israel Journal of Health Policy Research published seven papers [10–16] that related to different aspects of patient-centered care: the rights, roles, experiences, and perspectives of patients about their interactions in different health care settings. These articles underscore the difference between what clinicians think patients’ value and what they care about, reinforcing the critical importance of partnering with patients to understand their experiences of illness and what they hope for in encounters with a provider or the health care system.

Several of the recent IJHPR articles focused specifically on the principles of patient centered care described in the Picker Institute publication, *Through the Patient’s Eyes*, namely, respect for patients’ values, preferences and expressed needs; coordination and integration of care; information, communication and education; physical comfort; emotional support and alleviation of fear and anxiety; involvement of family and friends; continuity and transition; and access to care [1].

For example, Israel’s hospitals are required by the Ministry of Health to be accredited by Joint Commission International (JCI). Sperling and Pikkel [10] examine the patient and family rights that are highlighted in the JCI accreditation process and compare those with other Israeli regulations relating to patient and family rights. Building upon that, Carsten Engel from Denmark who has had extensive experience in reviewing hospital accreditation organizations in various countries, provides a perspective on how accreditation and legislation differ [11]. He observes that “they should not be seen as competing but as complementing efforts. Laws define minimum standards, whereas accreditation standards describe optimal performance; laws focus on the rights, whereas accreditation standards also point out ways in which hospitals may act to deliver these rights…”.

Moshe Flugelman and his colleagues address a very different aspect of patient-centered care – trust in the physician-patient relationship [12]. They studied patients who were referred to a hospital for coronary angiography, and found that the patients who had greater trust in their referring physician had less anxiety; and this trust in the referring physician had an even greater effect than did whether or not the patient had prior contact with the physician performing the procedure. The concept of how clinicians and organizations manage the “transfer of trust” is critical to a person’s wellbeing and to enhancing coordination of care. When patients are reassured that that the clinicians or staff involved in the next step of their care are excellent, stress is reduced, and the outcomes are typically improved.

Israel has a very high rate of in vitro fertilization (IVF) treatments, which is covered for all women under the national health insurance benefit. Infertility treatment can also be highly stressful. Medina-Artom and Adashi studied perceptions by IVF patients and providers in eight of Israel’s 25 IVF treatment units [13]. They examined multiple aspects of patient-centered care, including: accessibility of providers, provision of information and of explanations, communication skills of providers, patient involvement in the treatment, respect for patient values and needs, continuity and transition in treatment, professional competence, care organization, physical comfort, and emotional support. Although there was some room for improvement in all of aspects of care, a major finding was that in three areas – patients’ need for information, respect, and emotional support – “providers tended to underestimate the needs of fertility treatment patients”. Providers also gave themselves substantially higher scores than did patients on these aspects of patient-centered care.

This discrepancy between patient and physician perceptions is, unfortunately, common. Patient perceptions of their own health has been shown to have an association with health outcomes. Yet, a study of 33 U.S. family physicians and 506 of their patients found that the physicians and patients agreed on the state of the patient’s health less than 40% of the time [17]. Generally, patients tended to rate their health lower than did their physicians. The findings of the study indicated that the physician responses focused on determining the presence or absence of disease; whereas the patients focused their responses on their overall feeling of well-being and quality of life.

Interestingly and with similar findings, Berger et al. [14] performed a sophisticated study of Israeli physicians’ perceptions of the physician-patient relationship. They analyzed survey responses of almost 300 physicians and concluded, “In contrast to patients who traditionally stress the importance of interpersonal skills, physicians stress the significance of the technical expertise and knowledge of health providers, emphasizing the role of competence and performance. Physicians evaluate the relationship based on their ability to solve problems through devotion, serviceability, reliability, and trustworthiness and disregard the ‘softer’ interpersonal aspects such as caring, appreciation, and empathy that have been found to be important to their patients.”

The US-based Foundation for Informed Medical Decision-Making conducted several studies to identify concordance between physicians and patients on treatment of breast cancer to create decision aids and to understand the differences between what patients value and what their clinicians assume they value. In a study of values about post-mastectomy reconstructive surgery, patients placed greater importance than clinicians on avoiding use of a prosthesis (33% vs. 0, 95% CI of the difference: 13, 54). There was also a non-statistically significant trend toward less patient concern about “looking natural without clothes” compared to providers (24% vs. 40, 95% CI of the difference: − 12, 44) [18]. Patients were clearly more concerned about the practical value of reconstructive surgery versus the cosmetic value. It is easy to understand why having this knowledge should change the focus of discussions between surgeons and patients a great deal.

In a related study about factors that influence choice of a lumpectomy vs a mastectomy, patients were significantly less likely than providers to consider ‘keep your breast’ as a top goal when choosing surgery (7% vs. 71, 95% CI of the difference: − 92, − 37) when they understood that lumpectomy could result in higher rates of recurrence and the potential for more chemotherapy or surgery in the future; however, their surgeons assumed the only information that mattered was mortality outcomes [19]. These studies illuminate the need of health professionals to explore patient values and preferences to help identify the treatment options that best suit their needs and also to include the issues most paramount to patients when discussing treatment options.

Hayek et al. [15] studied the satisfaction of a national sample of Jewish and Arab Israelis with their primary care physicians. Although, as is generally the case in studies of patient satisfaction vs. patient experience, most survey respondents were very satisfied with the performance of their primary care physicians, there was an ethnic difference in the results. Arab patients reported lower satisfaction related to communication skills, interpersonal manners, and time spent by the physician. Hayek et al. appropriately recommended that better patient-centered results could be achieved by “improving the communication skills of the PCP, encouraging interpersonal interaction between the PCP and the patient, and devoting more time to the patient during the visits.” However, before assuming that these interventions will be successful, it would be helpful to conduct focus groups or interviews with Arab patients to gain a deeper understanding of the specific actions clinicians can take to improve these aspects of care. The results might be surprising, and they might be easier to implement than extensive communication training programs. These insights could help physicians become masters of both the art and the science of medicine.

Although interpersonal interactions are extremely important in providing patient-centered care, the Picker Principles also focus on making health care institutions and systems patient-centered. Bar-Lev and Beimel address one aspect of the systems approach in a paper that discusses the nature of lab test result reports that are accessible to patients via electronic patient portals [16]. They analyzed 225 patient responses to an online questionnaire and concluded that patients tended to overestimate the seriousness of the information “when it was presented either numerically or graphically compared to the narrative format.” They recommended that “graphs, tables, and charts would be easier to interpret if coupled with a brief verbal explanation; highlighting an overall level of urgency may be more helpful than indicating a diversion from the norm; and statements of results should include the type of follow-up required.”

Recognizing this, in one of the authors’ health systems, Boston’s MassGeneralBrigham, it is now commonplace to get input from patients and families about all patient-facing educational materials, communications, resources such as the patient portal. Diverse patient partners review these and frequently provide feedback that they are filled with jargon, written at a very high literacy level, and fail to address the important questions that patients have about the topic. The MassGeneralBrigham system has saved thousands of dollars through these reviews by creating meaningful communications that achieve the intended goal, and that do not end up in garbage cans or ignored.

Before closing, we would like to point out that this commentary consistently uses the term, and is about, patient-centered care. Furthermore, we have given the characteristics of this concept above. There are proponents of other terms and concepts that seem similar to patient-centered care, but, depending on the user, may be defined differently. These include “people-centered care” (see: https://healthstandards.org/general-updates/people-vs-patient-centred-care-whats-difference/), “person-centered care” [20], and “person-focused care” [21].

Patient-centered care was the subject of the 2017 Health System Leadership Conference sponsored by Israel’s National Institute for Health Policy Research and held at the Dead Sea. The recent IJHPR articles noted above are clearly a step in the right direction of improving patient-centered care. They call attention to the importance of engaging with patients and their families in many different ways – through shared decision-making between a patient and healthcare professional, through comprehensive public policy events and campaigns, and through engaging patients in self-management programs for chronic disease. Whatever form it takes, changing the focus from taking unilateral action to improve health and healthcare *for* the people, to taking action *with* the people is a simple, yet radical, notion. In 1998, at a Salzburg Global Summit on patient-centered care, Valerie Billingham, a fellow from the UK, suggested the motto for the patient-centered movement - “Nothing about me, without me.” We look forward to the day when this is routinely honored and when the clinical paradigm moves from only focusing on “What is the matter?” to “What matters to you?” [22, 23] There is an international movement to promote patient-centered care that currently involves 49 countries. For further information about this movement including how one can join it, see: https://wmty.world. Partnering with patients and their families in patient-centered care that matters will ensure that we deliver the care we all want and deserve.

## Data Availability

N/A.
